# Heterologous Expression and Bioactivity Determination of *Monochamus alternatus* Antibacterial Peptide Gene in *Komagataella phaffii* (*Pichia pastoris*)

**DOI:** 10.3390/ijms24065421

**Published:** 2023-03-12

**Authors:** Xu Chu, Di Jiang, Lu Yu, Ming Li, Songqing Wu, Feiping Zhang, Xia Hu

**Affiliations:** 1 Forestry College, Fujian Agriculture and Forestry University, Fuzhou 350002, China; 2 Key Laboratory of Integrated Pest Management in Ecological Forests, Fujian Agriculture and Forestry University, Fuzhou 350002, China; 3 International Joint Laboratory of Forest Symbiology, Fujian Agriculture and Forestry University, Fuzhou 350002, China

**Keywords:** *Monochamus alternatus*, antimicrobial peptide, heterologous expression, bioactivity

## Abstract

Insects have evolved to form a variety of complex natural compounds to prevent pathogen infection in the process of a long-term attack and defense game with various pathogens in nature. Antimicrobial Peptides (AMPs) are important effector molecules of the insect immune response to the pathogen invasion involved in bacteria, fungi, viruses and nematodes. The discovery and creation of new nematicides from these natural compounds is a key path to pest control. A total of 11 AMPs from *Monochamus alternatus* were classified into 3 categories, including Attacin, Cecropin and Defensin. Four AMP genes were successfully expressed by *Komagataella phaffii* KM71. The bioassay results showed that the exogenous expressed AMPs represented antimicrobial activity against *Serratia* (G^−^), *Bacillus thuringiensis* (G^+^) and *Beauveria bassiana* and high nematicide activity against *Bursaphelenchus xylophilus*. All four purified AMPs’ protein against *B. xylophilus* reached LC_50_ at 3 h (LC_50_ = 0.19 mg·mL^−1^ of MaltAtt-1, LC_50_ = 0.20 mg·mL^−1^ of MaltAtt-2 and MaltCec-2, LC_50_ = 0.25 mg·mL^−1^ of MaltDef-1). Furthermore, the AMPs could cause significant reduction of the thrashing frequency and egg hatching rate, and the deformation or fracture of the body wall of *B. xylophilus.* Therefore, this study is a foundation for further study of insect biological control and provides a theoretical basis for the research and development of new insecticidal pesticides.

## 1. Introduction

In the long process of biological evolution, in order to adapt to a variety of adverse environments and pathogen invasion, insects, as the most abundant and widely distributed species in the world, evolved a highly adaptive defense mechanism [1,2]. Insect innate immunity has played an important role in environmental adaptive evolution [3,4]. Antimicrobial Peptides (AMPs) were reported as important effector molecules of the insect immune response to the pathogen invasion involved in bacteria, fungi, viruses and nematodes [5,6,7]. As a fast-acting effector molecule, the induced antibacterial activity can last for several days and resist the invasion of pathogenic microorganisms [8].

Many insect AMPs have been identified, and of these, the most studied AMPs are Hymenoptera, with more than 30 AMPs identified; studies on insect AMPs of other insect orders, such as Coleoptera, are relatively rare [9]. The attacin isolated from *Spodoptera exigua* (Hübner) (Lepidoptera) has antibacterial activity against the Gram-negative bacteria *Escherichia coli* (Enterobacteriaceae: *Escherichia*) and *Pseudomonas chicory* (Pseudomonadaceae: *Pseudomonas*), and the Gram-positive bacteria *Bacillus Subtilis* (Bacillaceae: *Bacillus*) and *Listeria monocytogenes* (Listeriaceae: *Listeria*) [10]. Cecropin A from *Bombyx mori* (Lepidoptera, Bombycidae: *Bombyx*) shows high antifungal activity against the entomopathogenic fungus *Beauveria bassiana* (Cordycipitaceae: *Beauveria*), both in vitro and in vivo in silkworm larvae [11]. This suggests that AMPs commonly exist in insects and demonstrate a pivotal role in the innate immune system [12]. The composition of the antimicrobial peptide family also varies in different insects.

*Monochamus alternatus* Hope (Coleoptera: Cerambycidae) is not only a pest insect that can cause serious damage to *Pinus*, but also the main vector of pine wood nematode *Bursaphelenchus xylophilus* (Aphelenchoididae: *Bursaphelenchus*) in Asia, which causes pine wilt disease, a devastating disease of pine forests, and few pesticides can relieve this severe situation [13,14,15]. In the process of transmission, *M. alternatus* and *B. xylophilus* form symbiotic relationships. When the nematode enters the body, it may trigger the immune response and increase the secretion of natural compounds, including AMPs. The discovery and creation of new nematicides from these natural compounds is the key path to control pine wood nematodes, while biosynthesis is the first step in pest control using these natural products.

Compared with natural extraction from the insect hemolymph and chemical synthesis, the AMPs of heterologous expression have higher yield and purer products, and they compensate for the defect in protein inactivation by insufficient modification in post-transcriptional translation of AMP genes [16]. *Komagataella phaffii* (*Pichia pastoris*) (Phaffomycetaceae: *Komagataella*), as a eukaryotic organism, has strong resistance to AMPs, and the yield of *K. phaffii* is not reduced due to the inhibition of AMPs during mass cultivation, the most common heterologous expression system for AMPs [17,18,19,20,21].

In this study, the four AMP genes *MaltAtt-1*, *MaltAtt-2*, *MaltCec-2* and *MaltDef-1* from *M. alternatus* were identified, and then the recombinant expression vector of the pPIC9K-AMPS-specific peptide was constructed for heterologous expression. In addition, we revealed the resistance effects of four heterologous expressed AMPs to pathogenic microorganisms of *M. alternatus* and the inhibition of pine wood nematodes.

## 2. Results

### 2.1. Phylogenetic Analysis of M. alternatus AMP Genes

**Figure 1:**
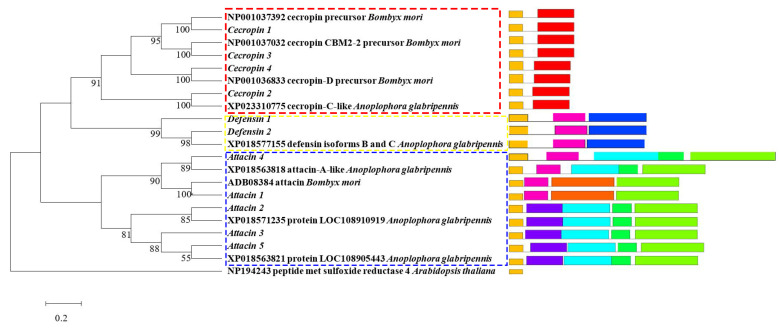
Phylogenetic analysis of *M. alternatus* AMP genes.

Phylogenetic analysis was performed on the obtained complete sequence of *M. alternatus* AMP genes, including five *Attacin* genes (*MaltAtt1*–*MaltAtt5*), four *Cecropin* genes (*MaltCec1*-*MaltCec4*) and two *Defensin* genes (*MaltDef1*, *2*) (Figure 1). The *M. alternatus* AMP genes had the highest consistency with *B. mori* and *Anoplophora*, and *Glabripennis* (Cerambycidae: *Anoplophora*) on the amino-acid sequences. *Attacin*, *Cecropin* and *Defensin* genes clustered well in different species, indicating that AMP genes of the same type were highly conserved.

### 2.2. Bioassays of AMPs

#### 2.2.1. Antimicrobial Activity Detection of AMPs

The recombinant plasmid was proved to be correctly constructed, and all four transformants were positive clones (Figure S1). The purified proteins were detected after methanol-induced expression for 48 h. The target bands were detected by SDS-PAGE and western blot of an anti-His tag antibody. The results showed that the AMPs of *M. alternatus* were successfully expressed by *K. phaffii* KM71 (Figure S2).

**Figure 2:**
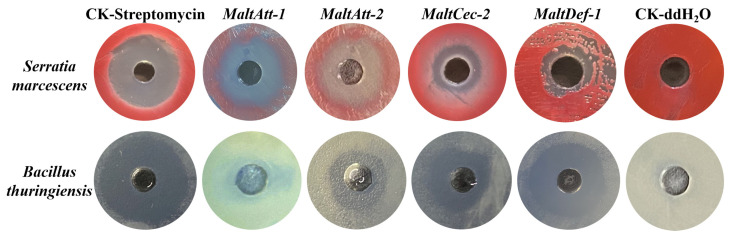
Bacteriostatic circles of *M. alternatus* AMPs protein.

**Figure 3:**
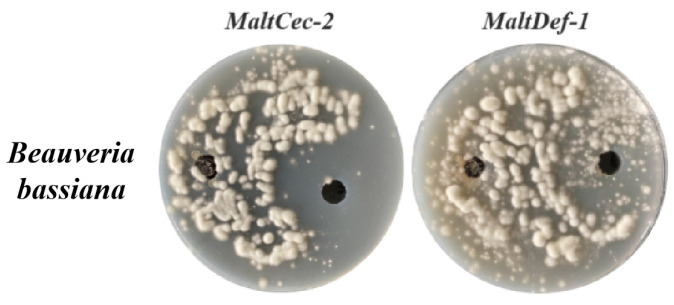
Antifungal circle of *M. alternatus* AMPs protein.

During the bioassays of expressed AMPs, MaltAtt-1 and MaltAtt-2 showed obvious antibacterial activity against the Gram-negative bacteria *Serratia marcescens* (Yersiniaceae: *Serratia*), with an inhibition circle diameter of 2 cm. Further, MaltCec-2 and MaltDef-1 showed significant antibacterial activity against the Gram-positive bacteria *Bacillus. thuringiensis* (Bacillaceae: *Bacillus*), with a diameter of 1.5 cm (Figure 2). ddH_2_O as a negative control had no obvious inhibition circle. Moreover, MaltCec-2 and MaltDef-1 showed obvious inhibitory effects on the fungi *B. bassiana*, while MaltAtt-1 and MaltAtt-2 showed no significant effect (Figure 3). These results indicated that the AMPs expressed in the *K. phaffii* eukaryotic expression system had antimicrobial activity.

#### 2.2.2. Hemolytic Activity Detection of AMPs

**Figure 4:**
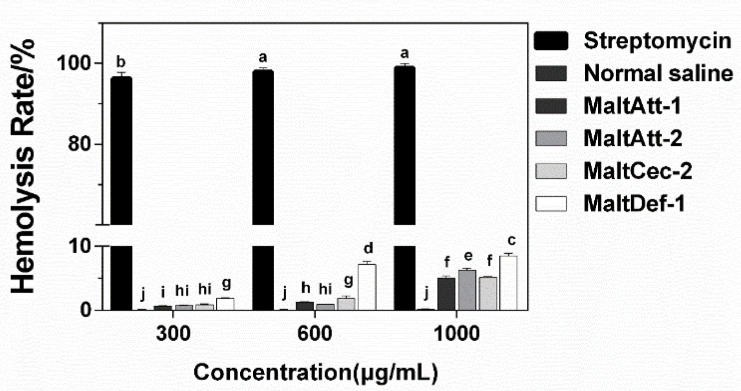
Hemolysis rate of *M. alternatus* AMPs. Different letters (a, b, c, etc.) above bars denote values that are significantly different from each other (*p* < 0.05, Tukey’s test).

According to the hemolytic test on animal cells, when the concentration of AMP is 1.0 mg·mL^−1^, the hemolysis rate of animal cells (rabbit blood cells) is 5–10%, and the hemolysis rate of streptomycin as a positive control group is above 90% (Figure 4), indicating that *M. alternatus* AMPs have low hemolytic activity on animal cells within the range of the bacteriostatic concentration. Among these four AMPs, the hemolysis rates of MaltAtt-1 (5.03%) and MaltCec-2 (5.13%) were significantly lower than those of MaltCec-2 (6.27%) and MaltDef-1 (8.5%) at the high concentration (1.0 mg·mL^−1^) (F = 18.14, df = 2, *p* < 0.05) (Figure 4).

#### 2.2.3. Bioassays with *B. xylophilus*

**Figure 5:**
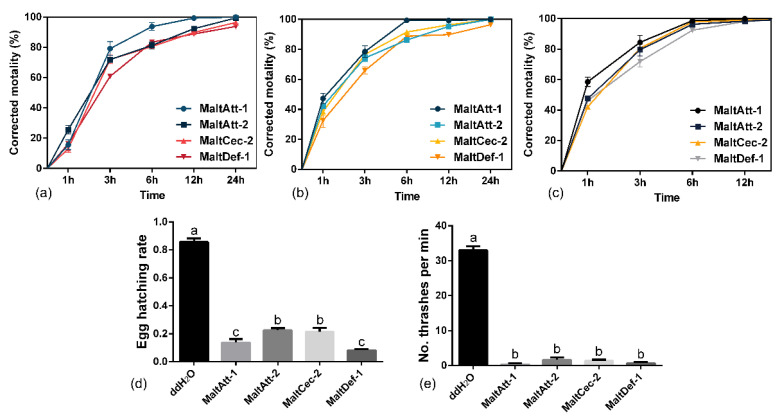
Bioassays with *B. xylophilus*. (**a**) Time–concentration–mortality trends of *B. xylophilus* treated with 0.3 mg·mL^−1^ purified AMPs; (**b**) Time–concentration–mortality trends of *B. xylophilus* treated with 0.6 mg·mL^−1^ purified AMPs; (**c**) Time–concentration–mortality trends of *B. xylophilus* treated with 1.0 mg·mL^−1^ purified AMPs; (**d**) Egg hatching rate; (**e**) Thrashing frequency (times per min). Different letters (a, b, c, etc.) above bars denote values that are significantly different from each other (*p* < 0.05, Tukey’s test).

The *M. alternatus* AMPs have a great toxicity effect on *B. xylophilus*. After treatment with *B. xylophilus* for 3 h, all 4 purified AMPs reached LC_50_ (LC_50_ = 0.19 mg·mL^−1^ of MaltAtt-1, LC_50_ = 0.20 mg·mL^−1^ of MaltAtt-2 and MaltCec-2, LC_50_ = 0.25 mg·mL^−1^ of MaltDef-1) (Figure 5a–c), and the toxicity activity of MaltAtt-1 was 98.7% at the low concentration of 0.3 mg·mL^−1^ after 6 h treatment. MaltDef-1 (83.4%) was second, followed by MaltAtt-2 (82.9%) and MaltCec-1 (80.9%) (Figure 5a). After treatment with *B. xylophilus* for 24 h, the toxicity activity of all 4 purified AMPs was 100.0% at 1.0 mg·mL^−1^ (Figure 5c). The purified AMPs significantly reduced the level of *B. xylophilus* egg hatching rates (F = 210.8, *p* < 0.0001) (Figure 5d) and thrashing frequencies (F = 485.7, *p* < 0.0001) (Figure 5e) compared to the nematodes treated with ddH_2_O. The egg hatching rates of *B. xylophilus* treated with MaltAtt-1 decreased from 85.7% to 13.7%. There was no significant difference between MaltAtt-1 and MaltDef-1 (8.2%) (t = 2.048, df = 4, *p* = 0.1099), while they were significantly lower than MaltAtt-2 (22.6%) and MaltCec-2 (21.5%) (*p* < 0.05) (Figure 5d). The thrashing frequency was also sharply reduced from 33 to 0.33 times per min. There was no significant difference among them (F = 1.905, *p* = 0.2073) (Figure 5e).

### 2.3. Effects of M. alternatus AMPs on Morphological Structure of B. xylophilus

**Figure 6:**
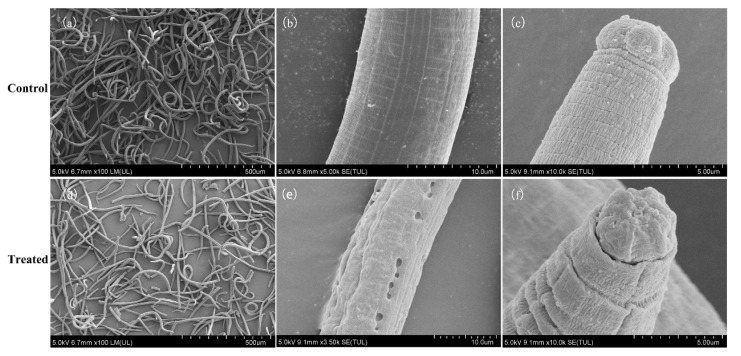
Observation by scanning electron microscope on morphologies of *B. xylophilus* treated with 0.3 mg·mL^−1^ purified AMP (MaltAtt-1). (**a**) normal *B. xylophilus*; (**b**) body wall of normal *B. xylophilus*; (**c**) head of normal *B. xylophilus*; (**d**) treated *B. xylophilus*; (**e**) body wall of treated *B. xylophilus*; (**f**) head of treated *B. xylophilus*.

Based on the bioassay results, MaltAtt-1 was selected for the morphological observation experiment. The morphological structures of nematodes changed significantly after they were treated with the purified AMPs. Observation by SEM indicated that the body of the controlled nematodes was plump and naturally curved (Figure 6a). The boundary between the head and the body was clear, and the grain of the body wall was clear (Figure 6b,c). However, the head and body of nematodes treated with AMP seriously shrunk, the grain of the body wall was fuzzy, the surface was uneven, and the body surface holes formed by invagination were observed (Figure 6d–f).

## 3. Discussion

Insects possess an innate immune system that protects them from attacks by various pathogenic microorganisms that would otherwise threaten their survival [22,23,24,25]. One of the important components of this defense system is AMPs [26,27,28]. Insects produce a large amount of AMPs as the first line of defense against bacteria, viruses, fungi or parasites [29,30,31,32]. In the present study, AMP genes were identified from *M. alternatus* larvae firstly, and they achieved exogenous expression in *K. phaffii*. All of the expressed AMPs showed an inhibitory effect on pathogenic microorganisms, including the bacteria *Serratia* and *B. thuringiensis*, the fungus *B. bassiana* and pine wood nematode. This suggested that *P. pastoris* was an effective expression system for the biosynthesis of natural product AMPs from *M. alternatus*.

At present, the research on nematicide for the control of pine wood nematode is still in the exploratory stage. Due to the adverse effect on the environment and the resistance, the application of chemical nematicide is seriously limited; therefore, the development of efficient and environmentally friendly natural nematicide is imminent. The purified AMPs significantly affected the vitality, egg hatching and morphological structure of *B. xylophilus* in bioassays. Especially for MaltAtt-1, it caused the egg hatching rates of *B. xylophilus* to decrease from 85.7% to 13.7% at concentrations of 0.3 mg·mL^−1^ for 24 h. The thrashing frequency was reduced from 33 to 0.33 times per min, which means AMPs have the potential to be a novel, environmentally friendly nematocidal agent.

AMPs were also reported to be the response molecules of innate immunity after the invasion of other nematodes [33,34]. When nematodes enter the insect, the pathogen-associated molecular patterns and pattern recognition receptors (PAMPs and PRR) are activated, and physiological and immune defenses are turned on [35,36,37,38]. The immune responses of vector insects to parasitic nematodes include the synthesis and secretion of AMPs and the melanism coating reaction [35,39]. Peña and her colleagues reported that AMPs gene expression in *Drosophila* was significantly increased after infection with *Xenorhabdus nematophila* (Enterobacteriaceae: *Xenorhabdus*) [40]. AMPs were detected in the hemolymph of *Drosophila suzukii* (Matsumura) (Diptera: *Drosophilidae*) carrying pathogenic bacteria called *Nematophiles* [41]. Current research progress further indicates that antimicrobial peptides are a kind of protein with the potential to become novel nematicide.

The profiles of *B. xylophilus* observed by scanning electron microscope were significantly changed after treatment with AMPs (MaltAtt-1), demonstrating obvious shrinkage of the body wall and some holes, which may be related to the membrane action mechanism of AMPs. AMPs have a variety of modes of action. Some studies believe that the antibacterial activity of AMPs is greatly related to the number of positive charges carried, which can combine with the cell membrane through the charge action, destroying the permeability of the membrane, leading to the lysis of cell membranes and the release of cellular contents, after which the cell cannot maintain osmotic pressure balance and dies [42,43]. Most of the AMPs that have been found to inhibit bacteria and fungi also have the ability to kill parasites, such as malaria, leishmaniasis and *Trypanosoma cruzi* (Trypanosomatidae: *Trypanosoma*) [44,45,46]. Some researchers have found that the mechanism of AMPs’ killing of parasites is very similar to that of inhibiting bacteria, both of which cause the pathogen’s death by interacting with cell membranes [47,48]. However, the mechanism of AMPs on *B. xylophilus* remains to be further studied.

## 4. Materials and Methods

### 4.1. M. alternatus Larvae Rearing

Larvae of *M. alternatus* were cultured on an artificial diet at a temperature of 26 ± 2 °C and humidity of 60 ± 10% in the Key Laboratory of Integrated Pest Management in Ecological Forests, Fujian Agriculture and Forestry University, Fuzhou, China (Table S1), with daily observation and regular diet supplementation.

### 4.2. Phylogenetic Analysis of M. alternatus AMP Genes

The *M. alternatus* AMP genes were identified by *M. alternatus* transcriptome (Accession Number PRJNA814348) with *A. glabripennis* genome (GenBank PRJNA 348318) and *B. mori* genome (InsectBase IBG00145). BLAST (Version 2.7.1) was used to perform annotation sequence alignments on the *M. alternatus* AMP gene and the other insect genome. HMMER (Version 3.0) was used to align the domain. Translation of the amino-acid sequences of the identified *M. alternatus* AMP genes was performed by DNAMAN (Version 6.0) software. NCBI Open Reading Frame Findao was used to predict the Open reading Frame (ORF) of the AMP genes and further align the characteristic domain of the obtained protein sequences. The domain motif structure diagram of the *M. alternatus* AMP family was drawn using MEME-ChIP (Version 5.4.1), and the conservative motif was labeled. The AMP evolutionary tree was constructed using MEGA (Version 5.0) software.

### 4.3. Construction of M. alternatus AMP Genes Eukaryotic Expression Vector

Based on the identified AMPs *MaltAtt-1*, *MaltAtt-2*, *MaltCec-2*, *MaltDef-1* gene sequence information, the *SnaB* I restriction site and start codon were added to the 5′ end, and the stop codon and *Not* I restriction site were added to the 3′ end (Figure 2). The target genes were named *pPIC9K-Att-1*, *pPIC9K-Att-2*, *pPIC9K-Cec-2* and *pPIC9K-Def-1*, and sequence syntheses were performed (Genecreate, Wuhan, China) (Table S2). Recombinant expression vectors were obtained and transformed into the *E*. *coli* Top 10.

### 4.4. Induction Expression and Purification of AMPs

The recombinant plasmids were linearized with *Sac* I enzyme and electroporated into *K. phaffii* KM71 by the Bia-Rad Gene Pulse Electroporator (Bia-Rad, Hercules, CA, USA). The transformation colonies were identified by PCR. The positive yeast transformants of *pPIC9K-Att-1*, *pPIC9K-Att-2*, *pPIC9K-Cec-2* and *pPIC9K-Def-1* were cultivated in Yeast Extract Peptone Dextrose (YPD) medium at 30 °C overnight, then we inoculated 5% of this culture into 50 mL Buffered Glycerol-complex (BMGY) medium at 200 rpm, at 30 °C for 24 h until OD_600_ was close to 3.0. Cells were harvested by centrifugation at 5000 rpm for 10 min at room temperature and resuspended to Buffered Methanol-complex (BMMY) medium to induce expression of the recombinant proteins. The resuspended culture was grown for 48 h by the addition of 1% methanol every 24 h, followed by centrifugation at 6500 rpm for 10 min, and the total proteins were extracted according to the Solarbio yeast total protein extraction kit. The crude antibacterial peptide proteins were purified by the Genescript His label purification kit, and the purification effect of the protein was detected by Tricine-SDS-PAGE. Western blotting was then performed to confirm successful expression and purification of the protein. Immunoblots were performed with mouse anti-His-tag monoclonal antibody, goat anti-mouse IgG (H + L) and HRP, and the positive bands on the resulting membrane were detected using an ultrasensitive ECL chemiluminescence kit (Sangon Biotech, Shanghai, China). The protein content was determined by the Bradford method.

### 4.5. Assays of AMPs

#### 4.5.1. Antimicrobial Activity Detection of AMPs

The agar cavity diffusion method was used to further verify the antimicrobial activities of AMPs, including MaltAtt-1, MaltAtt-2, MaltCec-2 and MaltDef-1 [49,50]. The Gram-positive bacteria *B. thuringiensis*, the Gram-negative bacteria *Serratia* and the fungus *B. bassiana* were stored in the Key Laboratory of Integrated Pest Management in Ecological Forests, Fujian Agriculture and Forestry University, and used as the experimental strains. The ddH_2_O as a negative control and 0.1 mg·mL^−1^ streptomycin as a positive control were used to determine the antimicrobial spectrum.

#### 4.5.2. Hemolytic Activity Detection of AMPs

The eukaryotic cell hemolytic test was used to evaluate the solubility of AMPs on hemocytes. Sterile rabbit blood with anticoagulant (Solarbio, Beijing, China) was selected, and 50 μL AMP dilution was added to each 500 μL hemocyte. The concentrations were successively diluted as follows: 0.3, 0.6 and 1.0 mg·mL^−1^, and 50 μL normal saline and 0.1 mg·mL^−1^ streptomycin (diluted with normal saline) were used as the negative control and positive control, respectively. All samples were incubated at 37 °C for 1 h, 2 h and 3 h, and then the supernatant was collected. The absorbance at 416 nm was determined on the Microplate Reader.

(1) $$\mathrm{Hemolysis}\;\mathrm{Rate}\left(\%\right)=\frac{{\mathrm{OD}}_{t}-{\mathrm{OD}}_{\mathrm{nc}}}{{\mathrm{OD}}_{\mathrm{pc}}-{\mathrm{OD}}_{\mathrm{nc}}}\times 100$$

where OD_t_: sample tube absorbance; OD_nc_: negative control tube absorbance; OD_pc_: positive control tube absorbance. The parallel test data of each group were counted to calculate the hemolysis rate.

#### 4.5.3. Bioassays with *B. xylophilus*

To estimate the toxicity effects of AMPs, the *B. xylophilus* mortality, hatching rate and thrashing frequency were assessed. All treatments were replicated three times.

Mortality assessment: 10 μL *B. xylophilus* suspension (100 adults/well) and AMPs were added to a 24-well culture plate and diluted with ddH_2_O until the final concentrations were 0.3, 0.6 and 1.0 mg·mL^−1^. The sterile water treatment was the control. The culture plates were placed in a Constant Temperature Incubator at 26 ± 2 °C. Then, the number of lives and deaths of *B. xylophilus* at 3 h, 6 h, 12 h and 24 h was observed and counted under the microscope. The mortality rate in the treatment group was corrected by the Abbott formula.

Hatching rate assessment: Newly laid eggs were collected 24 h after healthy nematodes had mated. Approximately 30 eggs were added to each well of 24-well tissue culture plates. Eggs were then treated with 100 μL of 0.3 mg·mL^−1^ purified AMPs at 25 °C for 24 h. The treatment with ddH_2_O was the control. The number of hatched juveniles was recorded and used to calculate the egg hatching rate.

Thrashing frequency: *B. xylophilus* adults were treated with 100 μL of 0.3 mg·mL^−1^ purified AMPs at 25 °C for 12 h in 24-well tissue culture plates (100 adults/well). The treatment with ddH_2_O was the control. In each well, 10 living adults were randomly selected for observation, and the number of thrashes per minute was recorded.

### 4.6. Observation of B. xylophilus by Scanning Electron Microscope (SEM)

A total of 10,000 pine wood nematodes were treated with 0.3 mg·mL^−1^ AMPs at 26 °C for 3 h, and nematodes in the controlled group were treated with distilled water in the same way. PWNs were washed with PBS (0.1 M, pH 7.4) for three times and then fixed with 2.5% glutaraldehyde overnight at 4 °C. The sample was removed from the glutaraldehyde; dehydrated with 50%, 70% and 100% ethanol gradient; and then removed and placed in a supercritical dryer, dried for about 1 h, removed, fixed on the sample table, sprayed gold and tested.

## 5. Conclusions

In this study, 11 AMP genes were identified, and 4 AMPs were successfully expressed by *K. phaffii* KM71. The expressed AMPs showed antimicrobial activity and a toxicity effect on pine wood nematode. MaltAtt-1 performed the best for inhibitory activity of *B. xylophilus*, with the lowest LC_50_ at 3 h (0.19 mg·mL^−1^). Furthermore, AMPs could cause a significant reduction of the thrashing frequency and egg hatching rate, and the deformation or fracture of the body wall of *B. xylophilus.*

## Supplementary Materials

The supporting information can be downloaded at: https://www.mdpi.com/article/10.3390/ijms24065421/s1.
