# Supplementary material for: Heterologous Expression and Bioactivity Determination of Monochamus alternatus Antibacterial Peptide Gene in Komagataella phaffii (Pichia pastoris)
Source: Int J Mol Sci. 2023 Mar 12;24(6):5421. doi: 10.3390/ijms24065421 (PMC10049621; doi:10.3390/ijms24065421)
Supplement: Supplementary file 1 [file ijms-24-05421-s001.zip › ijms-2233268-supplementary.pdf]

Table S1. The artificial feed formula for *Monochamus alternatus* larvae.

Artificial feed formula									
Bark	Xylem	Millfeed	shrimp shell powder	saccharose	sodium benzoate	sorbic acid	Yeast Extract	agar powder	H_2O
100 g	50 g	60 g	10 g	40 g	4 g	2 g	25 g	30 g	350 mL

Table S2. Primers for verification of AMP genes

Primer Name	Sequence	Primer Usage
Attacin-1-F	GCTACCGGAAGATTTGAATTCCCTAGGGCGGCCGCGA	Determination
Attacin-1-R	GCTACCGGAAGATTTGAATTCCCTAGGGCGGCCGCGA	Determination
Attacin-2-F	CTTACGTAATGAAATACTTTGTTTTCTTTGCCATGATGACAG	Determination
Attacin-2-R	GGACACGGAAGATTTGAATTCCCTAGGGCGGCCGCGA	Determination
Cecropin-2-F	CTTACGTAATGAATTTCTCAAGAGCTCTGTTTTACGTTTTCG	Determination
Cecropin-2-R	ATCGCTCACGGGAAAGAATTCCCTAGGGCGGCCGCGA	Determination
Defensin-1-F	CTTACGTAATGTTGATTCCAATTCAGTCAAAACTAGTTGCAA	Determination
Defensin-1-R	AATTCTAAGCGAGTTTGCATTTGTCGTGCGGCCGCGA	Determination

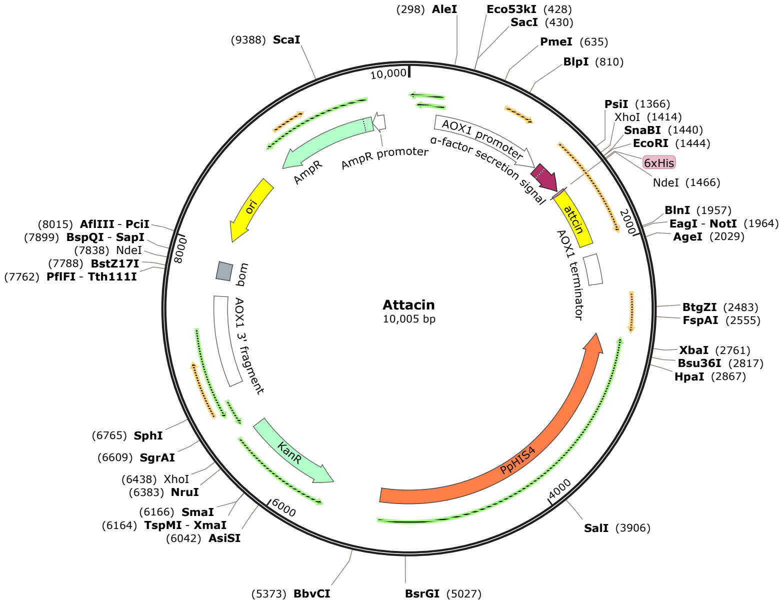

Figure S1. Map of AMP gene eukaryotic expression vector.

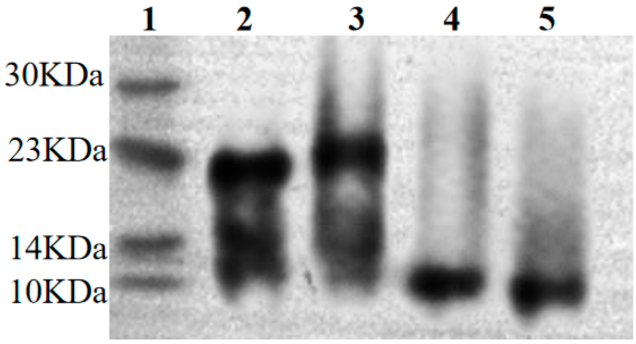

Figure S2. Immunoblot analysis of *M. alternatus* AMPs proteins. Lane 1: molecular weight markers; lane 2-5: *P. pastoris* induction expression product of pPIC9K-AMPs.
